# Chitosan membranes incorporating *Aloe vera* glycolic extract with joint synthesis of silver nanoparticles for the treatment of skin lesions

**DOI:** 10.1007/s13346-024-01683-x

**Published:** 2024-07-30

**Authors:** Venâncio A. Amaral, Victoria L. Santana, Erika S. Lisboa, Fredrico S. Martins, Marco V. Chaud, Ricardo L. C. de Albuquerque-Júnior, Wanessa Santana, Cochiran Santos, Adriana de Jesus Santos, Juliana C. Cardoso, Eliana B. Souto, Patrícia Severino

**Affiliations:** 1 Institute of Technology and Research, Tiradentes University, Murilo Dantas, 500, Aracaju, 49010-390 Sergipe Brazil; 2 Laboratory of Biomaterials and Nanotechnology, University of Sorocaba - UNISO, University City Campus, Raposo Tavares, Sorocaba, São Paulo, 18023-000 Brazil; 3 Post-Graduating Program in Dentistry, Department of Dentistry, Federal University of Santa Catarina, Florianópolis, 88040-370 Brazil; 4 Department of Pathology, Health Sciences Center, Federal University of Santa Catarina, Florianópolis, 88040-370 Brazil; 5 Laboratory of Pharmaceutical Technology, Department of Drug Sciences, Faculty of Pharmacy, University of Porto, Porto, 4050-313 Portugal

**Keywords:** Chitosan films, *Aloe vera*, Silver nanoparticles, Antimicrobial activity, Wound healing

## Abstract

New wound dressings based on polymeric membranes have been widely exploited for clinical applications to assist in the healing process and prevent additional complications (e.g., bacterial infections). Here we propose the development of a new production method of polymeric membranes based on chitosan, incorporating glycolic extract of *Aloe vera* with joint synthesis of silver nanoparticles for use as a new bioactive dressing. The membranes were obtained by casting technique, and their morphological, physicochemical characteristics, degree of swelling, degradation profile and antimicrobial activity evaluated. Morphological analyzes confirmed the synthesis and presence of silver nanoparticles in the polymeric membrane. The chemical compatibility between the materials was demonstrated through thermal analysis (TGA and DSC) combined with ATR-FTIR tests, showing the complexation of the membranes (Mb-Ch-Ex.Av-NPs). All membranes were characterized as hydrophilic material (with a contact angle (ө) < 90°); however, the highest degree of swelling was obtained for the chitosan. (Mb-Ch) membrane (69.91 ± 5.75%) and the lowest for Mb-Ch-Ex.Av-NPs (26.62 ± 8.93%). On the other hand, the degradation profile was higher for Mb-Ch-Ex.Av-NPs (77.85 ± 7.51%) and lower for Mb-Ch (57.60 ± 2.29%). The manufactured bioactive dressings showed activity against *Escherichia coli* and *Staphylococcus aureus*. Our work confirmed the development of translucent and flexible chitosan-based membranes, incorporating *Aloe vera* glycolic extract with joint synthesis of silver nanoparticles for use as a new bioactive dressing, with proven antimicrobial activity.

**Graphical Abstract:**

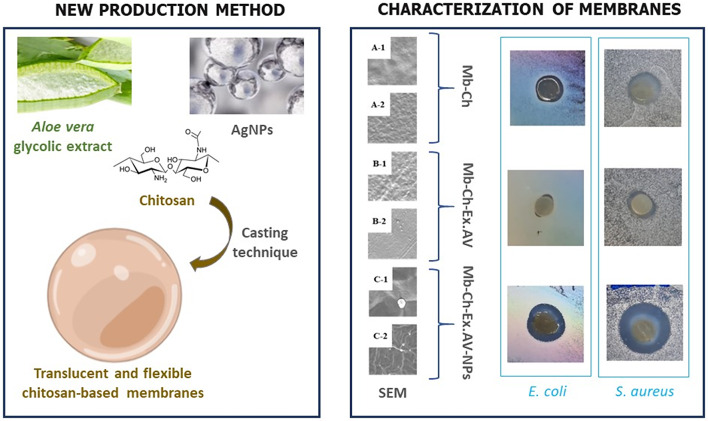

## Introduction

The skin is the largest organ in the human body and the first barrier against potential external agents harmful to the body. Furthermore, the skin helps to maintain the fluid homeostasis and body temperature, having unique immunological networks and neuroendocrine functions [1]. When injured, this organ can quickly initiate a tissue repair process to restore homeostasis, and a cascade of events to reestablish the barrier function, a process called healing [2–4].

The wound healing process represents a normal biological response of the human body however complex, involving biological, cellular and molecular processes, being achieved through four precise and highly programmed phases: (i) hemostasis, (ii) inflammation, (iii) proliferation, and (iv) remodeling [5]. For efficient wound healing, all these four phases must occur sequentially and at the appropriate time. However, several factors can negatively affect this process, such as infection, compromised blood circulation, presence of necrotic tissue, poor nutrition and advanced age, which can lead to chronic wounds and inadequate tissue repair [6, 7].

Unfortunately, every year, millions of people around the world are diagnosed with skin injuries, with an estimated number of ca. 37 million people suffering from chronic lesions. This alarming fact not only represents a disadvantage for the individual in terms of morbidity, disability and quality of life, but also affects the economy of countries, with prolonged treatments and hospital stays [8, 9].

Chronic wounds are defined by their failure to heal within the anticipated time frame. Over the past few decades, their occurrence has increased and the accompanying morbidity has come to light, making them an increasingly significant clinical problem. The treatment of chronic wounds relied on outdated standards of care, even a few years ago [9]. The most recent advances in the treatment of skin injuries are related to the technological processing of biomaterials in form of new devices (e.g., membranes, subdermal implants, dressings and nanoparticles), which act directly in healing and offer support for anchoring and transporting bioactive substances [10].

New wound dressings, such as polymeric membranes, have been widely studied for clinical applications to aid wound healing and prevent additional complications [11]. Membranes designed for use as a dressing must have a dense top layer to protect the wound from physical damage and infection, and have a porous and hydrophilic inner layer that allows the absorption of exudates, maintaining the appropriate environment for cell adhesion and proliferation, resulting in effective skin regeneration [12]. In view of this, scientific research is showing great interest in developing dressings using sustainable and economically viable materials, made from biomaterials, adopting synthetic routes that reduce the use of potentially toxic chemicals [13, 14].

Among commonly used biomaterials, chitosan and *Aloe vera* (L.) Burm.f. offer attractive biological and physicochemical properties for applications in dressings [13, 15, 16]. Chitosan is a mucoadhesiveness copolymer with well-established biocompatibility and biodegradability with a homeostatic effect, besides being non-toxic and non-antigenic [17, 18]. Furthermore, chitosan is capable of stimulating cell proliferation, affecting the function of macrophages, and thus accelerating the wound-healing process [19]. These properties are reinforced by the incorporation of bioactive compounds, such as *Aloe vera* extract; the exudate of this plant has been widely used to treat wounds [13]. *Aloe vera* is a medicinal plant used for centuries in countless cultures for various purposes, due to its complex constituents and diverse pharmacological activities [1]. This justifies its use, as the species has more than 75 potentially bioactives, which include minerals, amino acids, vitamins, enzymes, saccharides, lignin, salicylic acids, anthraquinones, and saponins [6].

As mentioned, the incorporation of other compounds is an effective way to enhance the properties of membranes when used as wound dressings. One possibility is the application of silver nanoparticles, a nanostructured system that has a broad spectrum of antibacterial (Gram-positive and Gram-negative bacteria), antifungal and antiviral properties [20, 21]. Together, these and other potential properties of each raw material can make polymeric membranes based on chitosan, *Aloe vera* glycolic extract and silver nanoparticles, a suitable dressing for treating wounds. The objective of the present work was to develop and characterize translucent polymeric membranes based on chitosan, incorporating the *Aloe vera* glycolic extract with joint synthesis of silver nanoparticles, with antimicrobial propertes for use as a potential wound dressing for skin lesions.

## Materials and methods

### Materials

Commercial chitosan (deacetylation degree of 86.7% and molar mass of about 1.47 × 10^5^ g/mol) was supplied by Polymar (Fortaleza, Brazil). *Aloe vera* glycolic extract (CAS Nr. 94349-62-9) was obtained from Farmalabor Raw Materials (Assago, Italy), with pH 3.56 (1:10), 1,030 − 1,060 g/ml density and 85–93% glycolic grade. Silver nitrate (molar mass 169.87 g/mol) was obtained from Dinâmica (São Paulo, Brazil) and sodium borohydride (molar mass 37.83 g/mol) was obtained from Neon (Suzano, Brazil). Glacial acetic acid (molar mass 60.05 g/mol) obtained from Synth (Diadema, Brazil) and glycerol (molar mass 92.09 g/mol) obtained from ACS Científica (Sumaré, Brazil) were used as acid medium and plasticizer, respectively. If not otherwise stated, all other chemicals and solvents of analytical grade were purchased from Sigma-Aldrich (St. Louis, MO, USA). Solutions were prepared with distilled or ultrapure water (home supplied).

### Preparation of material

#### Removal of impurities from Chitosan

Firstly, chitosan powder (2% w/v) was dissolved in an acetic acid solution (1% v/v) using a magnetic stirrer (Fisatom– Mod. 753 A, São Paulo, Brazil), for 24 h, at room temperature (25 ± 2 °C). After complete solubilization, the solution was centrifuged (Centrifuga DT 4000, DAIKI, São Paulo, Brazil) at 4000 ± 10 rpm for 15 min to remove possible impurities. The supernatant was collected to manufacture the membranes and the precipitate was discarded.

### Membrane fabrication

#### Preparation of polymeric membranes containing *Aloe vera* glycolic extract

The membrane formulations were prepared using the casting technique. Glycerol (1.5% w/v) was added to the impurity-free chitosan solution as a plasticizing agent and *Aloe vera* glycolic extract (3% v/v). The polymeric mixture obtained was stirred using a magnetic stirrer until complete homogenization. The resulting solution was poured into polyethylene Petri dishes (0.21 g/cm^2^) and dried in an oven (Tecnal, Mod– TE 393/2, São Paulo, Brazil) at 40 °C for 24 h or complete drying. The same methodology produced a control sample (membrane without *Aloe vera* glycolic extract).

#### Preparation of polymeric membranes containing *Aloe vera* glycolic extract and silver nanoparticles (AgNPs)

The membrane formulations were prepared using the casting technique. Glycerol (1.5% w/v) as a plasticizing agent, *Aloe vera* glycolic extract (3% v/v) and silver nitrate (5 mM in relation to the total volume of the polymeric mixture) were added to the impurity-free chitosan solution. Separately, a sodium borohydride solution (10 mM) was prepared as a silver reducing agent. The obtained polymeric mixture was kept under constant stirring using a magnetic stirrer, and a volume of 1500 µL of the reducing agent was added dropwise. The reaction was kept away from light with constant stirring, and the obvious change in the color of the reaction medium was an indicator of the formation of silver nanoparticles. At the end of the reaction, the resulting solution was poured into polyethylene Petri dishes (0.21 g/cm^2^) and dried in an oven at 40 °C for 24 h, or complete drying.

### Characterization techniques

#### Evaluation of thickness

Membrane thickness was recorded using a digital electronic micrometer (Electronic Digital Micrometer, Jomarca, Brazil), measuring in the range of 0–25 mm and precision of ± 0.001 mm). For each sample, 10 random points were analysed and registered, one point being in the center and the others on the perimeter (*n* = 10).

#### Fourier transform infrared spectroscopy (FTIR)

FTIR spectra were performed using Fourier Transform Infrared Spectroscopy equipment (FTIR– Cary 630 Agilent Technologies, Santa Clara, CA, USA). The chemical structures of the main functional groups of each molecule that make up the membranes were determined by Attenuated Total Reflectance (ATR) in the range between 4000 and 600 cm^–1^ at resolutions of 4 cm^–1^, with an average of 32 scans. The membrane samples were previously cut into a square shape (1 cm^2^) and kept in a desiccator with activated silica gel for 24 h.

#### Thermogravimetric analysis (TGA)

The thermogravimetric analysis of the membranes was obtained on a thermobalance (TGA55 Thermal Analyzer, TA Instruments, New Castle, DE, USA) in the heating range of 25–700 °C, with an inert nitrogen atmosphere, with a gas flow rate of 50 mL/min and at a rate of heating at 10 °C.min^–1^, in a platinum sample holder containing 2 mg of sample [22].

#### Differential scanning calorimetry (DSC)

Differential scanning calorimetry analysis was performed in a DSC 200 F3 Maia^®^ (NETZSCH, Selb, Germany). For this, the chitosan powder and membrane samples were analyzed by weighing 2 mg of sample into a hermetically sealed aluminum crucible and heated in an oven with an inert nitrogen atmosphere applying a flow of 50 mL.min^–1^, in the heating range of 25–350 °C, and a heating rate of 10 °C/min [22].

#### Scanning electron microscopy (SEM)

Analysis of the surface structure of the membranes was carried out using Scanning Electron Microscopy (SEM) (JSM-IT200, JEOL, Tokyo, Japan). The membranes were cut to 2 × 1 cm^2^ dimensions and kept in an environment with activated silica gel for 24 h. The membranes were fixed to an aluminum sample holder using double-sided carbon adhesive tape and electrically driven with a thin layer of gold through a metalizer, spraying for approximately 90 s at 3 mA. Scans were performed on the upper and lower parts of the membrane and sectioned surfaces [23].

#### Contact angle

The hydrophobic/hydrophilic properties of the membranes were evaluated by the contact angle in phosphate buffer saline (PBS) solution (pH 7.4). The contact angle of the PBS droplet under the surface of the samples was recorded using the LSA 100 Surface Analyzer (LAUDA Scientific GmbH, Lauda-Königshofen, Germany). For the measurement, a drop was added to the middle of the membrane, the contact angle was measured after 35 s using the Surface MeterTM software.

#### Degree of swelling

The membranes were analyzed for swelling capacity in phosphate buffer saline (PBS) solution (pH 7.4). Membrane samples with an area of 1 cm^2^ were weighed (Wd) and immersed in 3 mL of PBS, kept at a temperature of 37 ± 1 °C and were removed at intervals of 2 hours (times 0, 2, 4 and 6 h). After this period, the samples were removed and the excess of liquid remaining on the surface was removed using filter paper and weighed (Ww). The swelling capacity (%) was determined according to the equation represented below (Eq. 1) [23]:

1 $$\:Fluid\:uptake\:\left(\%\right)=\left(\frac{Ww-Wd}{Wd}\right)\:\times\:100$$

where Ww stands for the wet mass (g) of the samples at a certain time, and Wd for the initial mass (g) of the sample. Each sample was measured in triplicate.

#### In vitro degradation study

The in vitro degradation study followed the method described by Amaral et al. (2023) [24]. The samples were hydrated in phosphate buffer saline (PBS) solution (pH 7.4). to evaluate their degree of degradation. The membranes were cut into a square shape (1 cm^2^), weighed before the degradation study (Wd) and then immersed in 3 ml of PBS at 37 ± 1 °C for up to 120 h. The membranes were then removed at different times (24, 48, 72, 96 and 120 h), washed in distilled water to remove PBS salts and dried at 37 ± 1 °C until constant mass. Finally, the samples were weighed (Wa) and the percentage of weight loss was calculated according to the equation below (Eq. 2):

2 $$\:Weight\:loss\:\left(\%\right)=\:\left(\frac{Wd-Wa}{Wd}\right)\times\:100$$

where Wd stands for the initial mass of the sample (g), and Wa for the dry mass (g) of the samples at a specific immersion time. Each sample was measured in triplicate.

#### pH measurements

Membrane pH measurements were carried out using a benchtop digital pH meter (Kasvi, Pinhais, PR, Brazil), measurement accuracy of ± 0.02 pH) previously calibrated in the pH range between 4 and 7, at 25 ± 2°C. The membrane was cut in a square shape (1 cm^2^), weighed and then immersed in a in phosphate buffer saline (PBS) solution (pH 7.4). at 37 ± 1 °C for up to 120 h. As described, the membranes were removed at different times (24, 48, 72, 96 and 120 h) and the PBS was collected to evaluate the pH. The results were represented by the average of three measurements.

#### Antimicrobial activity

The microorganisms tested were standard strains of *Escherichia coli*– ATCC-25,922 (Gram-negative) and *Staphylococcus aureus*– ATCC-25,923 (Gram-positive). The evaluation of the antibacterial activity of the species was carried out using the disk diffusion method (Kirby-Bauer) in Mueller-Hinton agar culture medium (Kasvi, Pinhais, PR, Brazil). The polyethylene Petri dishes (90 × 15 mm) were divided into three areas to establish the spot to place the disc-shaped membranes (diameter 6 mm). Colonies of *E. coli* and *S. aureus* strains were prepared separately on Mueller-Hinton agar and diluted in saline solution (0.9% − 0.5 on the Mac-Farland scale) corresponding to approximately 2.10^8^ CFU/mL. Briefly, 200 µL of the microorganism suspension was spread on sterilized agar plates. After this procedure, the disc-shaped membranes were placed on the surface of the agar plates. The plates were then kept for 24 h, and incubated in an oven at 37 ± 1 °C to observe the inhibition zone.

#### Statistical analysis

The results were reported as the mean and corresponding standard deviation. The statistical significance of the differences of the analysed the parameters was assessed using ANOVA, followed by the Tukey test. A p-value of less than 0.05 indicates that the difference in results was considered statistically significant.

## Results and discussion

### Macroscopic characterization of polymeric membranes

**Fig. 1:**
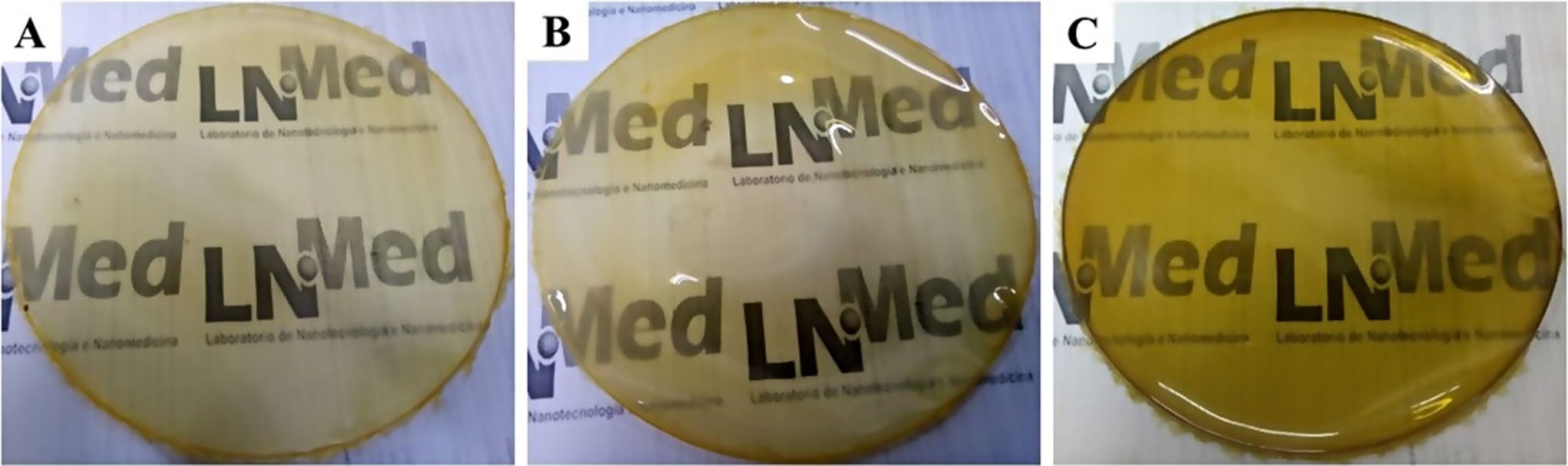
Macroscopic appearance of the developed polymeric membranes. **A**– Chitosan membrane (Mb-Ch); **B**– Chitosan membrane with *Aloe vera* glycolic extract (Mb-Ch-Ex.Av); **C**– Chitosan membrane with *Aloe vera* glycolic extract and silver nanoparticles (Mb-Ch- Ex.Av-NPs)

Macroscopic characterization of the membranes was carried out immediately after completing the drying process in an oven at 40 °C (Fig. 1). All the samples were transparent, homogeneous, flexible and with an excellent handling capacity. As seen in the images, the membranes appear translucent; however, exhibiting different rates of transparency depending on the combination of the raw materials used in their manufacturing process. This characteristic is extremely important, as the transparency of the membrane allows better monitoring of the wound healing process without removing the dressing [25–27].

Mb-Ch sample (Fig. 1A) generated a membrane with a light-yellow color. With the addition of *Aloe vera* glycolic extract (Fig. 1B), an increase in color intensity was observed. In the membrane containing the *Aloe vera* glycolic extract and silver nanoparticles, obtained with the aid of sodium borohydride (Fig. 1C), a change in color of the dispersion (from light-yellow to yellow-green) was observed during the synthesis of the membrane. This result confirms the reduction of silver ions (Ag^+^) to metallic silver (Ag^0^) with the formation of nanoparticles (AgNPs) [28].

The thickness of the membranes was measured using a digital electronic micrometer of 0–25 mm range and a precision of ± 0.001 mm, and the results are presented in Table 1.

**Table 1:** Assessment of the thickness of polymeric membranes. Chitosan membrane (Mb-Ch); Chitosan membrane with *Aloe vera* glycolic extract (Mb-Ch-Ex.Av); Chitosan membrane with *Aloe vera* glycolic extract and silver nanoparticles (Mb-Ch- Ex.Av-NPs)

Samples	Thickness (mm)
Mb-Ch	0.1781 ± 0.0099
Mb-Ch-Ex.Av	0.2545 ± 0.0741
Mb-Ch-Ex.Av-NPs	0.2929 ± 0.0275

The analyzed membranes had a diameter of ~ 9 cm (diameter corresponding to the Petri dish used as a container). When checking the structure of the polymeric membranes, a thinner thickness was shown for the Mb-Ch sample and a gradual increase in the average thickness of the samples with the addition of raw materials. An increase of 0.0764 mm was observed with the addition of *Aloe vera* glycolic extract (Mb-Ch-Ex.Av), and an increase of 0.1148 mm for the Mb-Ch-Ex.Av-NPs membrane when the extract was added and the synthesis of AgNPs was carried out.

The presence of glycolic extract of *Aloe vera* strengthened the internal bonds of chitosan, influencing the formation of hydrogen bonds between chitosan and glycerol during the membrane fabrication process. Consequently, the phenolic compounds and other constituents of the extract add mass to the membrane structure, resulting in an increase of its thickness. The incorporation of the glycolic extract of *Aloe vera* and AgNPs promotes a structural organization of the chitosan matrix. This organization can result in a more robust matrix, due to the interactions between the components, forming a more complex polymeric network and contributing to this increase in the thickness. These differences in membrane thickness were also proportional to the nature and composition of each sample, as shown in the present study (Table 1). This finding has also been reported by other researchers [29–31].

### Fourier transform infrared spectroscopy (FTIR)

**Fig. 2:**
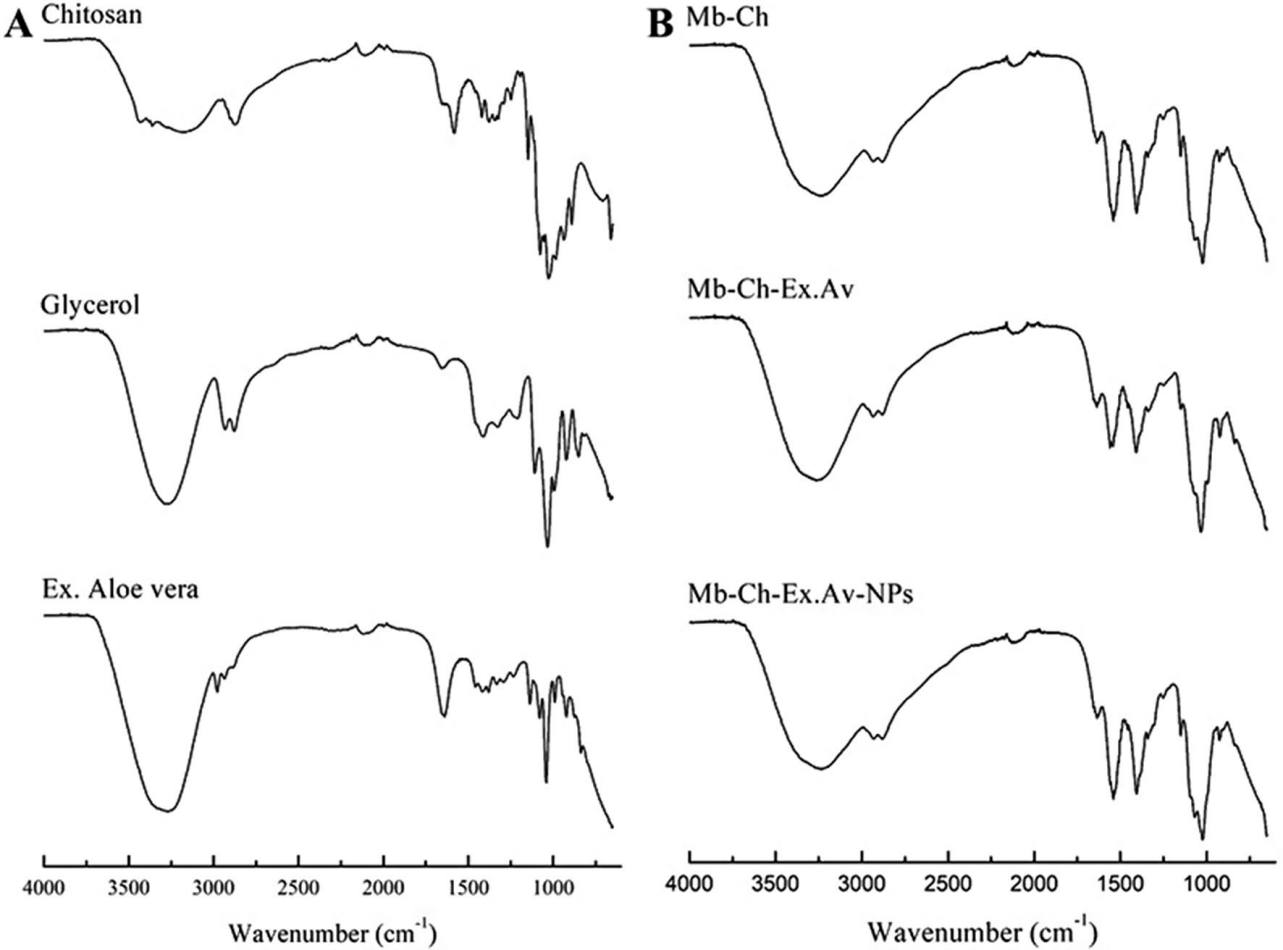
FTIR spectra of components (**A**) and polymeric membranes (**B**). Chitosan membrane (Mb-Ch); Chitosan membrane with *Aloe vera* glycolic extract (Mb-Ch-Ex.Av); Chitosan membrane with *Aloe vera* glycolic extract and silver nanoparticles (Mb-Ch-Ex.Av- NPs)

In order to study the chemical interactions between the components of the formulations, FTIR analyzes were carried out for the raw materials (chitosan, glycerol and *Aloe vera* glycolic extract) and for the polymeric membranes. The infrared spectra of the membrane components and samples are shown in Fig. 2(A, B).

FTIR spectra showed the characteristic chemical groups of the components (Fig. 2A). In the spectrum of chitosan, characteristic bands were observed at wavenumbers of 3240 cm^–1^ and 2877 cm^–1^, which are attributed to amide A and amide B. The structure of amide A arises mainly due to the stretching vibrations OH and NH. Amide B arises due to the stretching vibrations of the aliphatic bonds of the–CH group. Furthermore, the spectral bands observed at 1635 cm^–1^, 1543 cm^–1^ and 1340 cm^–1^ arise due to the C = O stretching vibrations, typical of amide I, NH bending vibrations, typical of amide II and the vibrations oscillating in the CH_2_ group, typical of amide III. The vibration for the COC group was confirmed by the peak at 1023 cm^–1^ [32].

Glycerol (Fig. 2A) is a chemical compound that contains OH and CH alkane groups in its structure. By observing the spectrum, it was possible to identify that the wavelength was between 3276 and 3630 cm^–1^, with a peak at 3286 cm^–1^, is attributed to the hydroxyl group (OH), while the CH stretch was identified by the band in the region of 2878–2932 cm^–1^ [33, 34]. Bands with typical transmittance of glycerol were also verified in the region known as the fingerprint (400–1500 cm^–1^). The band observed at 1410 cm^–1^ represents the bending of the COH group of the CO stretching of the primary alcohol, which is represented by the wavenumber of 1107 cm^–1^. The CO stretching of ether groups was observed at 1032 cm^–1^ and the bending vibration of the OH group at 921 cm^–1^ [35, 36].

In the spectrum of the *Aloe vera* glycolic extract (Fig. 2A), the represented bands ranged from the wavenumber of 3268 cm^–1^ to 835 cm^–1^. The characteristic bands were defined at 3268 cm^–1^ attributed to the presence of the hydroxyl group (OH), confirming the availability of phenolic compounds in the extract. The bands observed at 2116 cm^–1^ and in the range of 1640–1080 cm^–1^ suggest a correlation with the groups C = O, C = C, CH_2_ and CH_3_, showing the stretching vibration of aldehyde, aromatic ring, flavonoids, carboxyl, phenols and amino acids [37, 38]. The bands presented at 1041 cm^–1^ and 990 cm^–1^ can be referred to the presence of glycosidic bonds of saponin compounds [39].

According to the results presented by the FTIR analysis, the spectrum of the Mb-Ch membrane (Fig. 2B) presented a broad band between the wavenumber 3750–2980 cm^–1^ attributed to the presence of stretching of the hydroxyl group (OH), which overlap the stretching vibration of the amide A group in the same region. This fact may indicate the presence of chemical interactions, through hydrogen bonds, between the–NH group of chitosan and the OH group of glycerol. Another overlap was observed in the region of 2934–2885 cm^–1^, by the band present in glycerol, in relation to the amide B peak. At the peak at 1541 cm^–1^, attributed to amide II, an increase in intensity was observed in relation to isolated chitosan, this fact is due to the solubilization process in an acidic environment, indicating that the amino group is protonated (–NH_3_^+^ instead of–NH_2_). The formation of a new peak at 1404 cm^–1^ was also observed, attributed to the bending of the–CH_2_ group. Furthermore, the band in the region between 1121–936 cm^–1^, with an evident peak at 1023 cm^–1^, was observed with less distinct shoulder bands. All bands were assigned according to the literature [40–42].

The Mb-Ch-Ex.Av membrane showed changes compared to the Mb-Ch spectrum. When *Aloe vera* glycolic extract was added to the formulation, the spectrum showed a band with greater intensity in the region between 3750–2990 cm^–1^, corresponding to the OH group. This fact can be explained by the presence of phenolic compounds in the extract. In Counterpoint, a significant reduction in amide II intensity was observed at 1550 cm^–1^. This event may indicate that the substances present in the *Aloe vera* glycolic extract are interacting more effectively with the protonated group (–NH_3_^+^) of chitosan.

For the membrane incorporating AgNPs in the membrane (Mb-Ch-Ex.Av-NPs) it was possible to observe an opposite effect when compared to nanoparticles-free membrane (Mb-Ch-Ex.Av). A reduction in band intensity was observed in the region between 3740–2993 cm^–1^, corresponding to the OH group, and an increase in the intensity of the peak at 1550 cm^–1^, corresponding to amide II. Although sodium borohydride was used as a reducing agent, the synthesis of AgNPs was carried out in the polymeric mixture, thus, the use of plant extract also helped in this reduction process and stability, as it contains a wide variety of active substances in its composition (e.g., phenolic compounds) [43, 44]. This explains the change observed in the OH band and amide II, demonstrating a greater tendency of the OH terminals in relation to Ag^+^ ions and their conversion into Ag^0^ atoms, as opposed to the–NH_3_^+^ group. Another possibility lies in the complexation mechanism of AgNPs formed with the polymeric mixture [45].

### Thermogravimetric analysis (TGA)

Thermogravimetric analysis is an instrumental technique used to study the thermal decomposition, stability and composition of materials under controlled temperature conditions. The thermogravimetric curves generated during the TGA analysis may also show, in addition to the thermal stability of the material, its oxidative stability, multicomponent composition, product lifetime, decomposition kinetics, moisture and volatile content [46].

**Fig. 3:**
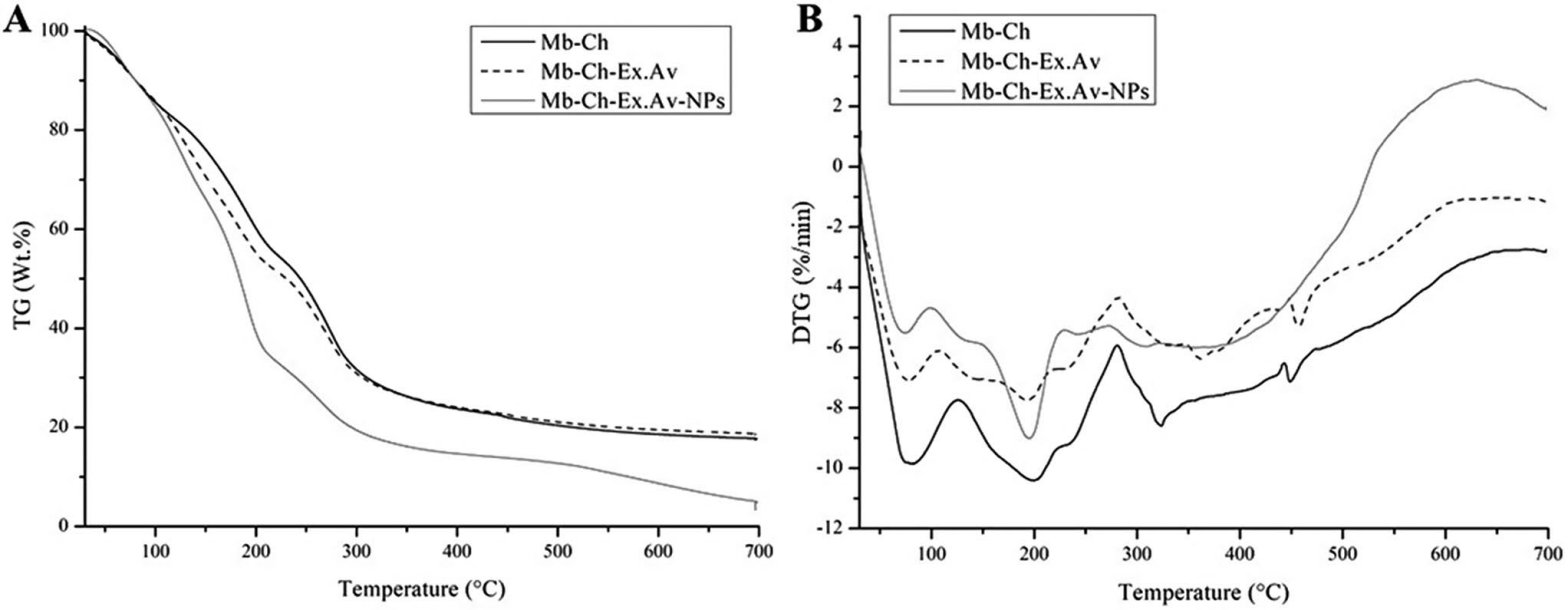
Analysis of TGA (**A**) and DTG (**B**) of polymeric membranes. Chitosan membrane (Mb-Ch); Chitosan membrane with *Aloe vera* glycolic extract (Mb-Ch-Ex.Av); Chitosan membrane with *Aloe vera* glycolic extract and silver nanoparticles (Mb-Ch-Ex.Av-NPs)

From the thermogravimetric curves (TGA and DTG) presented in Fig. 3(A, B), it is possible to observe a similar thermal degradation behavior, with two well-defined thermal events in the Mb-Ch, Mb-Ch-Ex.Av and Mb-Ch-Ex.Av-NPs samples.

The thermal degradation profile of the Mb-Ch membrane started from 30 to 99.8 °C, with a weight loss of 15%, attributed to the volatilization of moisture or solvent residue existing in the membrane. The second thermal event was recorded between 99.8 and 230.7 °C, with a weight loss of 32%, attributed to a complex process involving the decomposition of acetylated and deacetylated chitosan units and the glycerol structure [40, 47, 48]. Above 230 °C until the end of the study at 700 °C, a weight loss of 36% was recorded; in addition, at the end of the thermal analysis, a residual ash content equivalent to 17% of the sample was observed.

With the addition of *Aloe vera* glycolic extract, an increase in the initial decomposition temperature range was observed between 30 and 109.7 °C, a relative increase of 9.9 °C, with a weight loss of 17%. This change is related to the presence of phenolic compounds in the extract. In the second thermal event, an exponential increase was observed in the temperature range, reaching 281.2 °C, with a weight loss of 48%. This fact is due to the denaturation of the polymeric organization formed by chitosan and the plant extract [49]. Above 280 °C until the end of the study at 700 °C, a weight loss of 17% was recorded; in addition, at the end of the thermal analysis, a residual ash content equivalent to 18% of the sample was observed.

Notably, the addition of glycolic extract of *Aloe vera* modified the initial decomposition temperature range of the membrane, extending it to 109.7 °C. This is associated with the presence of phenolic compounds and indicates greater volatilization of these structures. The addition of the extract also resulted in a decrease in the maximum thermal degradation rate compared to Mb-Ch.

This phenomenon was also reported in the fabrication of nanofiber membranes based on *Aloe vera* extract, chitosan, pullulan, and citric acid [6]. The membranes were obtained using Forcespinning^®^ technology. As the content of *Aloe vera* extract increased, the membranes began to show a slight decrease in the maximum thermal degradation rate. Additionally, a slight increase in the residual mass percentage was observed due to the extract content [6].

In another report from the literature, electrospinning was used to create nanofibers with the inclusion of natural *Aloe vera* skin extract in poly(ethylene oxide)/PEO solutions [50]. The incorporation of different concentrations (5, 10, and 20% by weight) of *Aloe vera* in the PEO matrix resulted in a decrease in the thermal stability of the nanofibers, in terms of the onset decomposition temperature, being more evident at higher concentrations (10 and 20% by weight) [50].

**Fig. 4:**
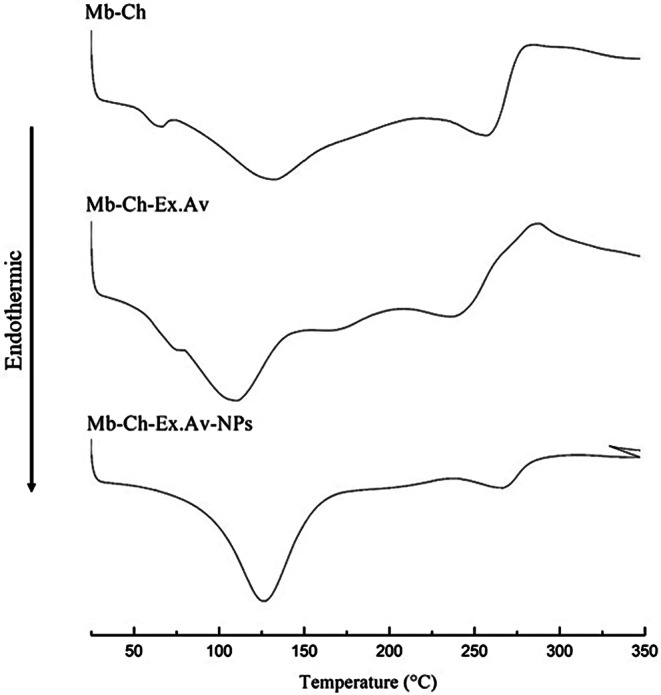
Thermoanalytical DSC curves of the developed membranes. Chitosan membrane (Mb-Ch); Chitosan membrane with *Aloe vera* glycolic extract (Mb-Ch-Ex.Av); Chitosan membrane with *Aloe vera* glycolic extract and silver nanoparticles (Mb-Ch-Ex.Av- NPs)

In the Mb-Ch-Ex.Av-NPs (the sample containing AgNPs), an increase in the initial decomposition temperature range up to 125.9 °C and also in weight loss of 25% was observed. These increases can be justified by the presence of phenolic compounds in the extract, which also help in the reduction process and stability of the nanoparticles. However, in the second decomposition event, no increase in the temperature range was observed, remaining at 281.2 °C, with a slight increase in weight loss to 53%. In the final section up to 700 °C, a weight loss of 19% was recorded. Furthermore, a large reduction was recorded in the residual ash content of 3% of the sample. This result can be explained by the degree of organization of the compounds in the polymer mixture, as observed in the FTIR spectrum (Fig. 2B) and in the DSC thermogram (Fig. 4). This demonstrates that the presence of phenolic compounds and AgNPs promotes a structural reorganization of the chitosan matrix, significantly reducing the residual ash content and indicating a more complete decomposition of the polymer matrix at high temperatures.

### Differential scanning calorimetry (DSC)

DSC is a fundamental tool used for thermal analysis, very useful to study the phase changes caused by temperature in various materials, from polymers to foods and pharmaceutical products. Furthermore, DSC can directly measure transitions such as melting, crystallization, and glass transition, providing qualitative and quantitative information about thermodynamic parameters, such as melting temperature (T_m_), glass transition temperature (T_g_), crystallization temperature (T_c_), and other thermal properties, such as heat capacity (C_p_) and melting enthalpy (ΔH) [51, 52]. Figure 4 presents the DSC analysis profiles for the manufactured Mb-Ch, Mb-Ch-Ex.Av and Mb-Ch-Ex.Av-NPs.

In the thermoanalytical curve of the Mb-Ch sample, three well-defined thermal events were observed, the first event at 61.41 °C (on set: 48.42 °C and endset: 71.75 °C), attributed to the glass transition temperature (T_g_), characteristic of membranes obtained using acetic acid as a solvent [40, 53]. The second event was observed at 132.39 °C (on set: 76.23 °C and endset: 210.64 °C) and is related to the loss of water or solvent residues in the membrane and the third event at 262.21 °C (on set: 228.76 °C– endset: 282.20 °C), marks the beginning of the complex thermal decomposition of the acetylated and deacetylated chitosan units and the glycerol structure, as shown in the TG analysis (Fig. 3).

With the addition of *Aloe vera* glycolic extract, changes were seen in the thermal events shown in the thermoanalytical curve of the Mb-Ch-Ex.Av sample, with an increase in the T_g_ temperature of 61.41 °C (Mb-Ch) for a temperature of 74.77 °C, reinforcing the chemical interactions that occurred between the components of the extract with the polymeric mixture also seen in the FTIR spectrum. In the second thermal event, a change to 111.21 °C was observed (on set: 80.81 °C– endset: 150.80 °C); this reduction can be attributed to the presence of volatile compounds in the extract, which are evaporated together with water or solvent residue. Above the temperature of 213.30 °C, an endothermic event was observed at 244.17 °C followed by an exothermic event at 286.77 °C, characteristic of the thermal decomposition of the mixture components, including the denaturation of the chemical structures present in the *Aloe vera* glycolic extract [49].

With the formation of AgNPs in the polymeric mixture of chitosan and *Aloe vera* glycolic extract, changes in the thermoanalytical curve of the Mb-Ch-Ex.Av-NPs sample were also recorded. Only two well-defined thermal events are presented, the first at 125.71 °C (on set: 44.30 °C and endset: 179.08 °C) related to the loss of volatile compounds from the extract, water or solvent residues, and the second at 268.63 °C (on set: 241.57 °C and endset: 298.02 °C), displaying the beginning of the complex thermal decomposition of the membrane components. What caught attention in the thermogram was the absence of Tg, reinforcing the hypothesis of the formation of a new remodeling of the spatial chemical structure, through the complexation mechanism of AgNPs with the polymeric mixture, resulting in a structure with a greater degree of organization.

### Scanning Electron Microscopy (SEM)

**Fig. 5:**
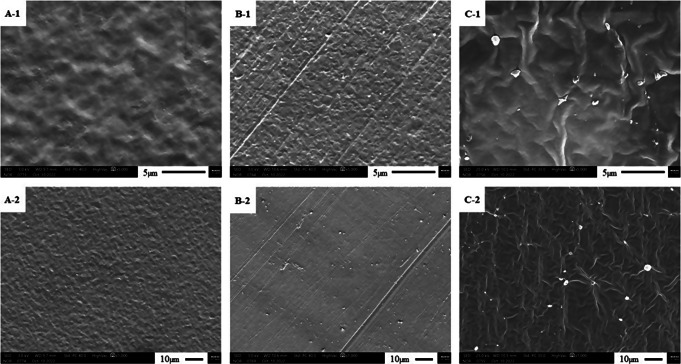
Scanning electron microscopy (SEM) analysis of Mb-Ch (**A 1–2**), Mb-Ch-Ex.Av (**B 1–2**) and Mb-Ch-Ex.Av-NPs (**C 1–2**). Chitosan membrane (Mb-Ch); Chitosan membrane with *Aloe vera* glycolic extract (Mb-Ch-Ex.Av); Chitosan membrane with *Aloe vera* glycolic extract and silver nanoparticles (Mb-Ch-Ex.Av- NPs)

The characterization of the membranes’ microstructure by SEM provides important information about the morphology of each polymeric membrane and the distribution of the different components used in the manufacturing process. The surface morphological characteristics of the Mb-Ch, Mb-Ch-Ex.Av and Mb-Ch-Ex.Av-NPs were investigated by SEM and are presented in Fig. 5.

Analyzing the surface morphology of the Mb-Ch sample (Figs. 1, 2 and 5A), it was possible to confirm the homogeneity of the polymeric mixture after the drying process, indicating coherence and good miscibility between the components. This sample depicts a compact morphology and without any visible cracks, however, with an irregular surface along its length, but without the presence of agglomerates or apparent formation of pores [42, 54].

In the SEM of the membrane containing *Aloe vera* glycolic extract (Mb-Ch-Ex.Av, Figs. 1, 2 and 5B), the homogeneity of the polymeric mixture and a more regular surface structure were also observed, however, with the presence of small agglomerates along its length, but without the formation of pores.

When analyzing the surface morphology of the membrane incorporating AgNPs (Mb-Ch-Ex.Av-NPs, Figs. 1, 2 and 5C), greater amounts of agglomerates and irregularities were observed on the surface of the material, generating a surface structure with a rough appearance, but without the formation of pores. These morphological characteristics may be associated with the chemical interactions formed during the AgNPs synthesis process with the polymeric mixture.

### Contact angle measurement

**Fig. 6:**
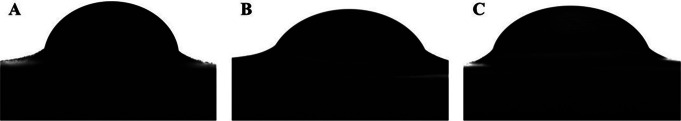
Contact angle and wettability of polymeric membranes. **A**– Chitosan membrane (Mb-Ch); **B**– Chitosan membrane with *Aloe vera* glycolic extract (Mb-Ch-Ex.Av); **C**– Chitosan membrane with *Aloe vera* glycolic extract and silver nanoparticles (Mb-Ch-Ex.Av- NPs)

The contact angle is a measurement that allows evaluating the degree of wettability of materials, highlighting characteristics of hydrophobicity or hydrophilicity in the face of the interfacial interaction between a liquid and a solid [55]. Wettability is an important property for practical membrane applications, such as biological dressings. In fact, a dressing must have an adequate capacity to maintain a moist environment for the healing process, and/or to avoid excessive dehydration and the accumulation of exudate [56]. The membrane contact angle values are shown in Fig. 6; Table 2. Table 2 presents the contact angle measurements between the liquid (PBS pH 7.4) and the polymeric membrane samples.

**Table 2:** Contact angle measurement of polymeric membranes. A– Chitosan membrane (Mb-Ch); B– Chitosan membrane with *Aloe vera* glycolic extract (Mb-Ch-Ex.Av); C– chitosan membrane with *Aloe vera* glycolic extract and silver nanoparticles (Mb-Ch-Ex.Av- NPs)

Samples	Contact angle (°)
(A) Mb-Ch	88.53 ± 1.42
(B) Mb-Ch-Ex.Av	68.05 ± 0.15
(C) Mb-Ch-Ex.Av-NPs	70.30 ± 0.35

Material surfaces can be classified as superhydrophilic (ө < 10°), hydrophilic (ө < 90°), hydrophobic (ө > 90°) and superhydrophobic (ө > 150°) [55]. According to the results, the membrane with the highest contact angle, i.e., with the lowest interfacial interaction between the liquid and the solid, was the Mb-Ch sample with 88.53 ± 1.42°. This finding is in agreement with the literature [41], since the chemical structure of chitosan, as well as the structure of glycerol, has a hydrophilic characteristic due to the presence of OH functional groups, causing a reduction in the contact angle below the hydrophobic range.

With the addition of *Aloe vera* glycolic extract (Mb-Ch-Ex.Av), a reduction in the contact angle was recorded, amounting to 68.05 ± 0.15°, which can be attributed to the hydrophilic components present in the extract. However, when AgNPs were incorporated into the polymeric mixture (Mb-Ch-Ex.Av-NPs), the contact angle value showed a slight increase up to 70.30 ± 0.35°. Based on the contact angle results, it is possible to state that all manufactured membranes have an angle below 90°, being all classified as hydrophilic material.

### Analysis of the degree of swelling

**Fig. 7:**
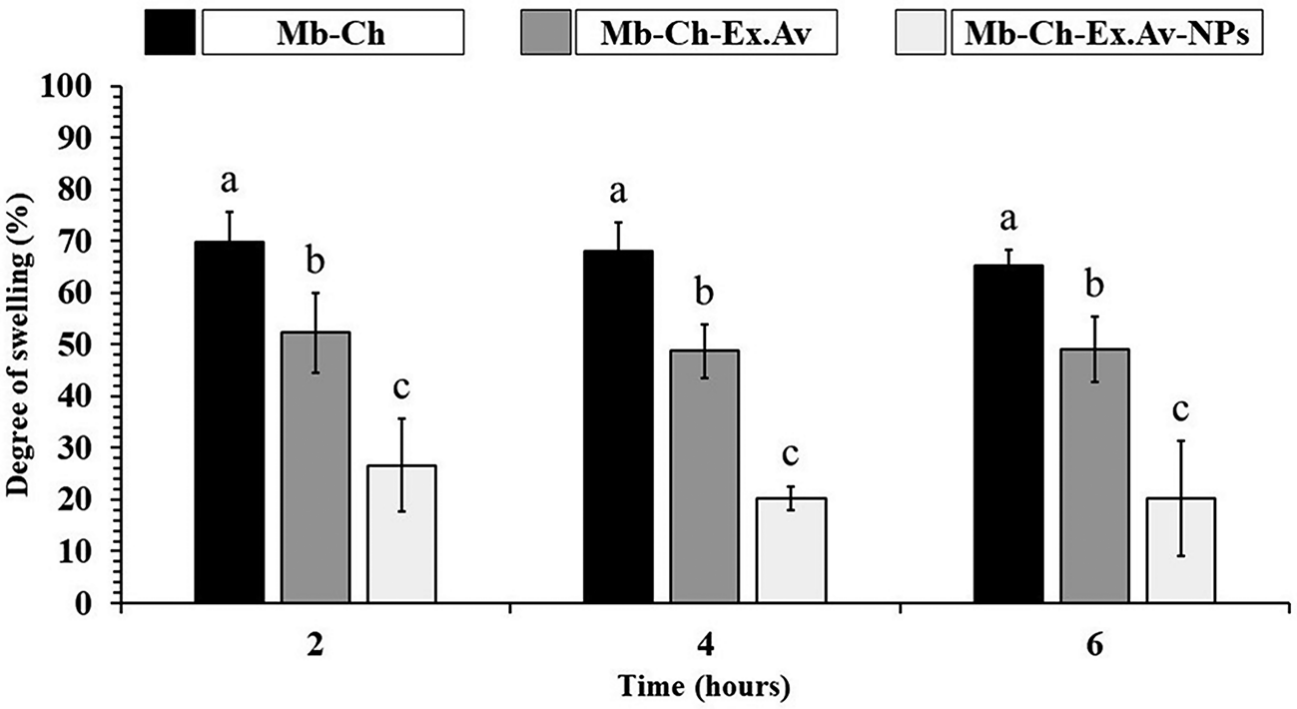
Fluid absorption of polymeric membranes in PBS (pH 7.4 at 37 ± 1°C). Chitosan membrane (Mb-Ch); Chitosan membrane with *Aloe vera* glycolic extract (Mb-Ch-Ex.Av); Chitosan membrane with *Aloe vera* glycolic extract and silver nanoparticles (Mb-Ch-Ex.Av- NPs)

The swelling profile of each polymeric membrane was evaluated using phosphate buffer saline (PBS) solution (pH 7.4) at 37 ± 1 °C. Figure 7 shows the swelling behaviour of the developed membrates, related to the fluid absorbed and retained by the entire membrane structure at times 2, 4 and 6 h of contact.

By analyzing the membrane swelling results, a higher percentage of fluid absorption for Mb-Ch (69.91 ± 5.75%) was recorded, a fact observed at all time points. This occurrence is related to the strong hydrogen bond between the functional groups of chitosan and water molecules [57].

Due to the manufacturing method in an acidic medium, Mb-Ch exhibits a structure with protonated functional groups (–NH_3_⁺). These groups are hydrophilic and can form hydrogen bonds with water molecules. Additionally, the positive charges formed along the structure mutually repel each other, and this electrostatic repulsion also contributes to swelling by creating spaces in the polymer chains, allowing the entry of more water molecules. This result is consistent with the literature, where a chitosan-based membranes showed water absorption of 65%, justified by the presence of hydrophilic functional groups in the membrane structure [58]. Another study in the literature reported the swelling behavior of membranes based on *Aloe vera* gel and chitosan crosslinked with different crosslinking agents (PVA/Glutaraldehyde/Tween80) in various phosphate buffer media [59]. The swelling behavior of the membranes was altered in different pH media, due to the protonation/deprotonation of functional groups, highlighting the importance of the structures present in the polymeric mixture, and how these groups can significantly modulate the degree of membrane swelling [59].

In our study, with the addition of *Aloe vera* glycolic extract to chitosan-based membranes, a significant reduction in swelling capacity was observed, especially when AgNPs were synthesized together with the polymeric mixture. Compared to Mb-Ch, the Mb-Ch-Ex.Av exhibited a swelling capacity of 52.33 ± 7.71%, with the presence of AgNPs in the Mb-Ch-Ex.Av-NPs resulting in a significant decrease, recording a value of 26.62 ± 8.93%.

The reduction shown by Mb-Ch-Ex.Av can be explained by the increase of chemical interactions between the chitosan matrix and the plant extract, indicating that the substances present in the *Aloe vera* glycolic extract promoted interactions with polar groups of the polymers, reducing the availability of these groups to interact with water molecules [54, 60].

The drastic reduction presented by Mb-Ch-Ex.Av-NPs is related, not only to the crosslinking of the chitosan matrix and the plant extract, but also to the complexation of AgNPs by the polymeric mixture. A similar result was observed by Thompson et al. [61], for PVA membranes containing silver particles. In that study, silver functionalization reduced the swelling capacity of the samples. This was explained by the effect of incorporating Ag^+^ ions, which replace water molecules and subsequently interact with the electron donors of iminodiacetic acid. In response to this reaction, the water content is reduced in the functionalized membranes [61].

Another study described the development of chitosan nanocomposite membranes incorporated with sulfonated titanium oxide (TiO₂) [58]. With the incorporation of the inorganic load, the sulfonated TiO₂ nanoparticles caused a decrease in the water absorption of the membranes. Due to the interactions between the–SO₃H groups of the sulfonated TiO₂ and the functional groups of chitosan, water absorption in the membranes is decreased. The incorporation of sulfonated TiO₂ nanoparticles also limits the mobility of the chitosan chains and the water storage sites, reducing water absorption in the membranes [58].

Based on these results, we can suggest that the presence of AgNPs, formed during the membrane fabrication process, promotes a greater reorganization of the polymeric matrix. The AgNPs interact with the chitosan matrix and the plant extract, resulting a more complex and robust polymeric network. This nanoparticle complexation mechanism with the membrane results in a less permeable structure, filling the available spaces in the polymeric matrix, which further hinders the absorption of water molecules.

### In vitro degradation study

**Fig. 8:**
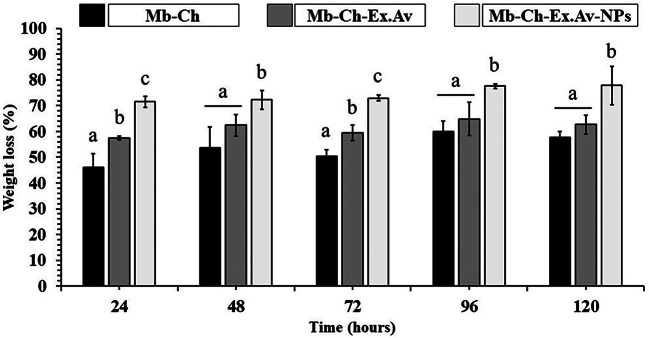
Weight loss (%) of the developed polymeric membranes, in PBS (pH 7.4) at 37 ± 1 °C. Chitosan membrane (Mb-Ch); Chitosan membrane with *Aloe vera* glycolic extract (Mb-Ch-Ex.Av); Chitosan membrane with *Aloe vera* glycolic extract and silver nanoparticles (Mb-Ch-Ex.Av- NPs)

The rate of degradation of a membrane for use as a dressing is a critical factor during the skin heating and regeneration process, as it is the basis for remodeling and morphogenesis to form functional tissue [56]. The in vitro degradation study of Mb-Ch, Mb-Ch-Ex.Av and Mb-Ch-Ex.Av-NPs was monitored as a function of incubation time at 37 ± 1 °C, as shown in Fig. 8.

The analysis of the results shows that the mass loss rate (%) of Mb-Ch and Mb-Ch-Ex.Av samples, after 120 h of incubation, did not show a statistically significant difference (*p* > 0.05), with values of 62.69 ± 3.65% and 57.60 ± 2.29%, respectively. Regarding Mb-Ch-Ex.Av-NPs sample, the outcome was more evident, showing a degradation of 77.85 ± 7.51% (*p* < 0.05). In detail, the variation in the mass ratio between the first point (24 h) and the end point (120 h) was 11.58%, 5.21%, 6.28% for the Mb-Ch, Mb-Ch-Ex.Av and Mb-Ch-Ex.Av-NPs, respectively. The degradation results demonstrate that the manufactured membranes have long-term stability. Controlled degradation is a characteristic of interest, mainly to avoid unnecessary clinical interventions that could harm tissue recovery. Furthermore, as membranes degrade into smaller fragments, they not only create spaces (pores) for tissue growth, but also allow the controlled release of active substances [52]. In this way, components of the *Aloe vera* glycolic extract are released into the injured tissue, such as glucomannan and gibberellin, substances responsible for the healing properties due to increased collagen production in and around the wound area [62].

### 10. Measurements of pH

**Fig. 9:**
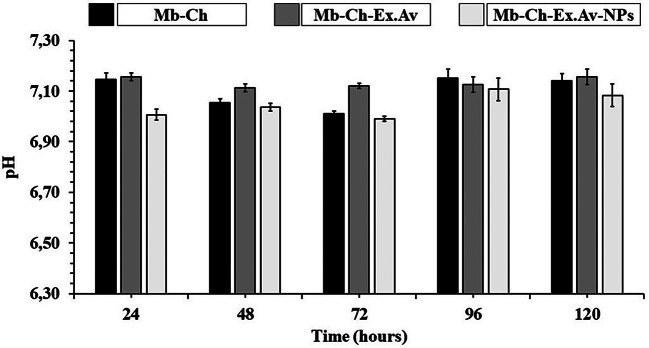
Results of pH measurements of the developed polymeric membranes in PBS (pH 7.4) at 37 ± 1 °C. Chitosan membrane (Mb-Ch); Chitosan membrane with *Aloe vera* glycolic extract (Mb-Ch-Ex.Av); Chitosan membrane with *Aloe vera* glycolic extract and silver nanoparticles (Mb-Ch-Ex.Av-NPs)

During the in vitro degradation study of polymeric membranes, the pH values of each sample were recorded after incubation periods in PBS (pH 7.4) at 37 ± 1 °C. The recorded values are presented in Fig. 9.

During the in vitro degradation study, the generation of membrane fragments through hydrolytic reactions resulted in an imbalance of the pH of PBS, presenting a reduction in the pH scale at all times analyzed. The membrane samples presented a pH range between 7.01 and 7.15 (Mb-Ch), 7.11–7.16 (Mb-Ch-Ex.Av) and 6.99–7.11 (Mb- Ch-Ex.Av-NPs) throughout the 120 h of the study. These changes observed during degradation must be considered when aiming to use membranes as dressings in the management of wound healing, since the pH in the wound area varies throughout the healing process.

Healthy skin typically has a pH range of 4–6. On the other hand, purulent wounds and those containing necrotic tissue and crusty crusts typically have an average pH value of around 6.1, while chronic or infected wounds usually have a pH between 7 and 8 [52]. In this way, the reduction in pH by the fragments released by the membrane would act positively on the healing process, being a promising strategy for wound treatment.

### Assessment of antimicrobial activity

**Fig. 10:**
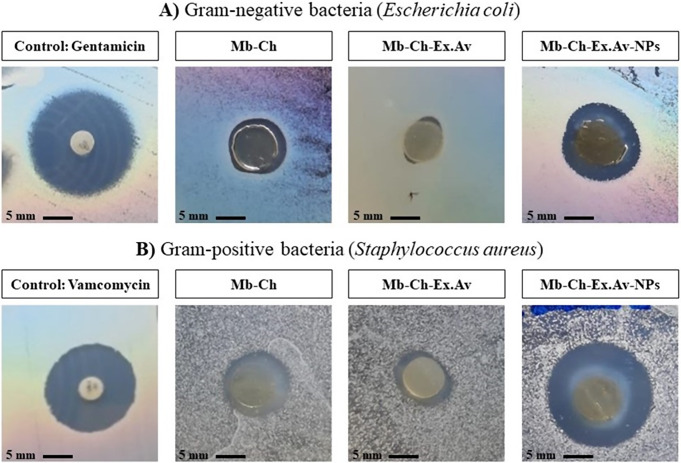
Disc diffusion test of polymeric membranes in strains of *Escherichia coli* (**A**) and *Staphylococcus aureus* (**B**). Chitosan membrane (Mb-Ch); Chitosan membrane with *Aloe vera* glycolic extract (Mb-Ch-Ex.Av); Chitosan membrane with *Aloe vera* glycolic extract and silver nanoparticles (Mb-Ch-Ex.Av- NPs)

The in vitro analysis of the antimicrobial activity aimed to visualize an inhibition halo of the tested microorganisms by the developed membranes. To evaluate the disk diffusion test, analysis was carried out on the Mb-Ch, Mb-Ch-Ex.Av and Mb-Ch-Ex.Av-NPs samples, investigating the inhibition of *E. coli* (Gram-negative) bacteria and *S. aureus* (Gram-positive). The results are presented in Fig. 10.

Plant extracts obtained from *Aloe vera* have shown effective antibacterial activities, suppressing the growth of Gram-positive and Gram-negative bacteria, due to the presence of bioactive compounds, such as anthraquinones, saponins, quercetin and catechin [62]. The inhibition of the growth of the tested microorganisms showed antibacterial activity with the formation of the halo (Fig. 10). Using these results, the diameters of each inhibition halo were measured and are shown in Table 3.

**Table 3:** Inhibition halo of polymeric membranes against strains of *Escherichia coli* and *Staphylococcus aureus*. Chitosan membrane (Mb-Ch); Chitosan membrane with *Aloe vera* glycolic extract (Mb-Ch-Ex.Av); Chitosan membrane with *Aloe vera* glycolic extract and silver nanoparticles (Mb-Ch-Ex.Av- NPs)

Samples	Inhibition halo (mm) E. coli	Inhibition halo (mm) S. aureus
Gentamicin (Control)	17.90 ± 0.14	-
Vancomycin (Control)	-	15.50 ± 0.71
Mb-Ch	9.50 ± 0.71	11.50 ± 0.71
Mb-Ch-Ex.Av	6.75 ± 0.35	9.60 ± 1.41
Mb-Ch-Ex.Av-NPs	13.30 ± 0.42	18.20 ± 0.28

From the obtained results, it is possible to see that the Mb-Ch-Ex.Av had the smallest inhibition halos for tested both strains. In relation to the *E. coli* strain, the formation of inhibition halos was observed for Mb-Ch (9.50 ± 0.71 mm) and for Mb-Ch-Ex.Av-NPs (13.30 ± 0.42 mm); however, these results are smaller when compared to the selected control (Gentamicin), 17.90 ± 0.14 mm.

When analyzing the results in relation to the S. aureus strain, we see the formation of inhibition halos of 11.50 ± 0.71 mm for the Mb-Ch sample. Furthermore, in this test with Gram-positive bacteria, the Mb-Ch-Ex.Av-NPs showed an excellent result, with an inhibition zone of 18.20 ± 0.28 mm, a diameter above the halo presented by the selected control (Vancomycin ) of 15.50 ± 0.71 mm.

The obtained results reaffirm the potential added value of chitosan-based membranes as dressings for the treatment of wounds, as they are biodegradable, safe, biocompatible and with antibacterial effects [63]. Interestingly, the Mb-Ch-Ex.Av led to a smaller inhibition zone compared to Mb-Ch; this fact can be explained by the contact time during the study (24 h). Based on the results presented in the degradation profile (Fig. 8), we can suggest that the contact time indicates the controlled release of the substances present in the *Aloe vera* glycolic extract, resulting in greater antibacterial activity.

Another fact observed in our study was the greater prevalence of inhibition in relation to Gram-positive bacteria. These findings are in agreement with the literature, where plant extracts exhibit stronger antibacterial activity against Gram-positive bacteria when compared to Gram-negative bacteria [64, 65].

Finally, the synthesis of AgNPs together with the polymeric mixture to manufacture the Mb-Ch-Ex.Av-NPs sample showed different inhibition rates depending on the analyzed strain, with *S. aureus* being more susceptible than *E. coli*. This difference may be related to the diversity in the composition and structure of the cell wall between Gram-positive and Gram-negative bacteria. Studies in the literature have already shown that nanoparticles synthesized from *Aloe vera* extract have a great antimicrobial efficiency against Gram-positive bacteria (*Staphylococcus aureus*,* Bacillus Subtilis*) and Gram-negative bacteria (*Klebsiella pneumoniae Escherichia Coli*) [43, 66–68]. AgNPs synthesized from plant extracts have proven to be effective antibacterial agents due to their size, shape and capping obtained during green synthesis [66]. Our data demonstrate that the composition of the polymeric membrane (Mb-Ch-Ex.Av-NPs) has effective antibacterial activity potential, in addition to assisting in the healing process when used as a wound dressing.

## Conclusions

The casting technique proved to be efficient in producing polymeric membranes for use as wound dressings. The chitosan used as the base polymeric matrix for the membranes was efficient for the incorporation of the *Aloe vera* glycolic extract and the synthesis of silver nanoparticles. With the incorporation of these components, the morphological and morphometric characteristics of the membranes could be modulated. The spectra obtained by FTIR showed chemical interactions between the polymers, highlighting the complexation mechanism of AgNPs with the chitosan structure and with the substances present in the *Aloe vera* glycolic extract. TGA and DSC thermal analyzes reaffirmed the occurrence of chemical interactions, promoting a new conformation of the spatial chemical structure of the membranes, with emphasis on Mb-Ch-Ex.Av-NPs, which presented a greater degree of organization in relation to Mb-Ch and Mb-Ch-Ex.Av. With the incorporation of *Aloe vera* glycolic extract and the synthesis of AgNPs, the membranes showed a lower swelling profile, namely, Mb-Ch-Ex.Av-NPs (26.62 ± 8.93%), Mb-Ch-Ex.Av (52.33 ± 7.71%) and Mb-Ch (69.91 ± 5.75%), respectively. On the other hand, the membranes showed a higher degradation profile when the content of *Aloe vera* glycolic extract and the synthesis of AgNPs were introduced in the process, being Mb-Ch-Ex.Av-NPs (77.85 ± 7.51%) > Mb-Ch-Ex.Av (62.69 ± 3.65%) > Mb-Ch (57.60 ± 2.29%), respectively. Regarding the antimicrobial activity of the membranes, inhibition against Gram-negative and Gram-positive bacteria was confirmed, however, the inhibition efficiency was greater for Gram-positive bacteria (*Staphylococcus aureus*), mainly for the Mb-Ch-Ex.Av-NPs. Based on the findings of this work, we conclude that chitosan-based membranes have the ability to incorporate *Aloe vera* glycolic extract and silver nanoparticles through the process of complexation which, to our knowledge has not been described yet. In addition, the manufacturing process enhances the inherent qualities of each raw material, while creating a new membrane that may be used as a dressing with both healing and antibacterial properties. Proof of concept in triple co-culture involving the simultaneous growth of dermal fibroblasts, keratinocytes, and either macrophages or T cells, will be helpful in assessing the efficacy of our innovative membranes, before reaching preclinical models.

## Data Availability

The datasets generated during and/or analysed during the current study are available from the corresponding author on reasonable request.
